# Supplementary figures and images for: Functional drug–target–disease network analysis of gene–phenotype connectivity for curcumin in hepatocellular carcinoma
Source: PeerJ. 2021 Oct 26;9:e12339. doi: 10.7717/peerj.12339 (PMC8555505; doi:10.7717/peerj.12339)

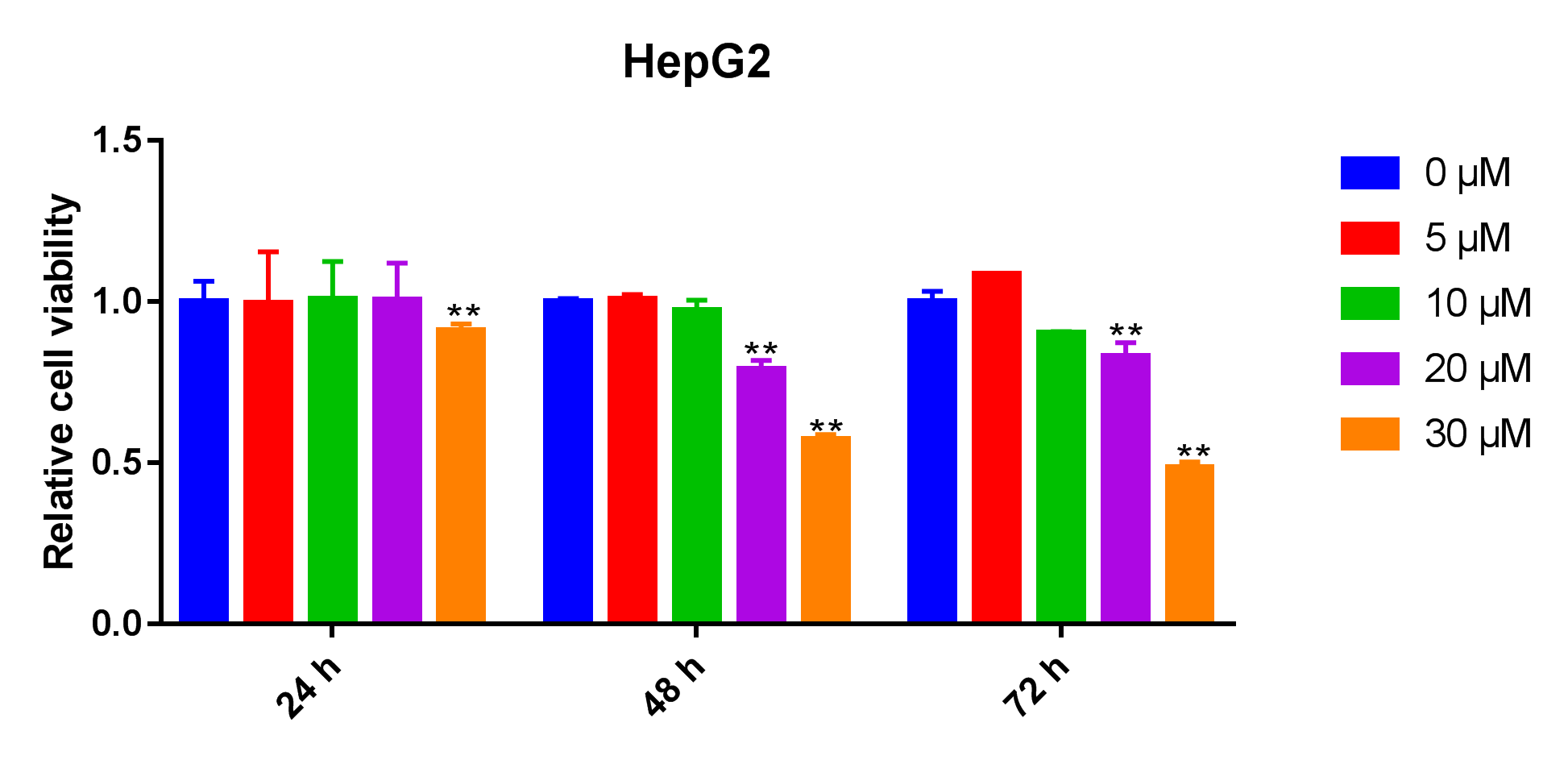

Supplement: Supplemental Information 2 [file peerj-09-12339-s002.zip › Raw data materials/Raw data for figure 4A/HepG2.tif]

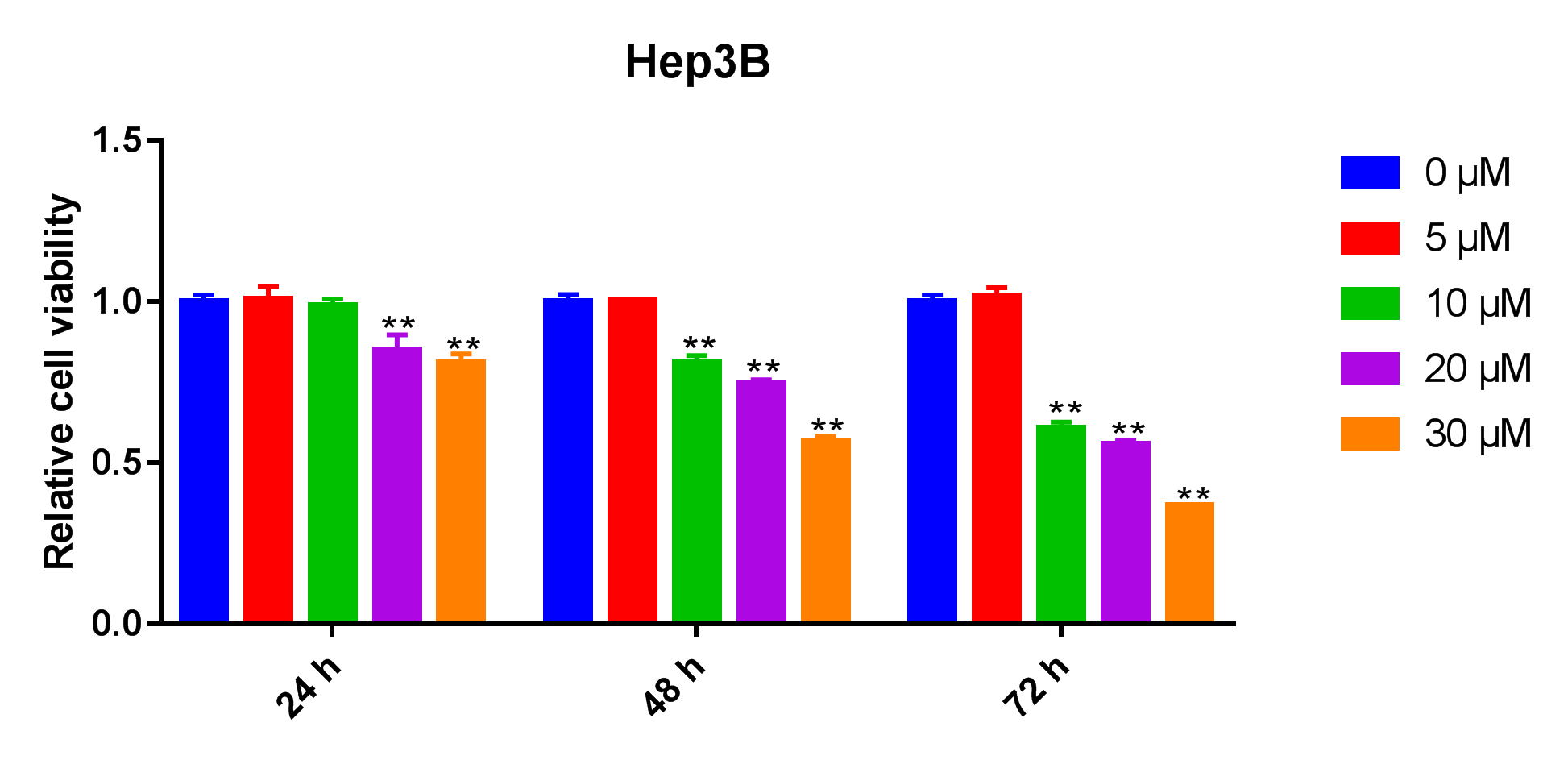

Supplement: Supplemental Information 2 [file peerj-09-12339-s002.zip › Raw data materials/Raw data for figure 4B/Hep3B.tif]

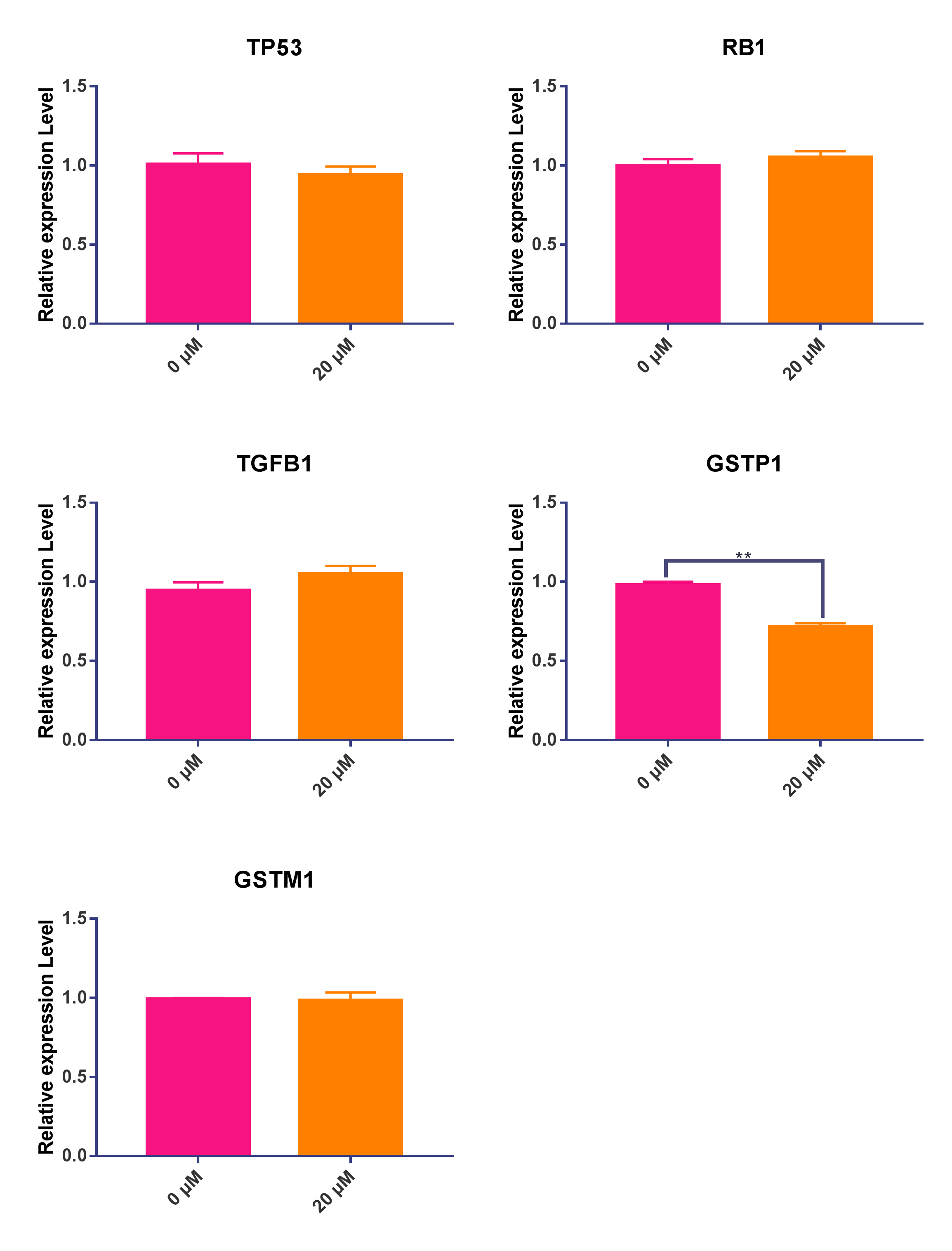

Supplement: Supplemental Information 2 [file peerj-09-12339-s002.zip › Raw data materials/Raw data for figure 4C/HepG2.tif]

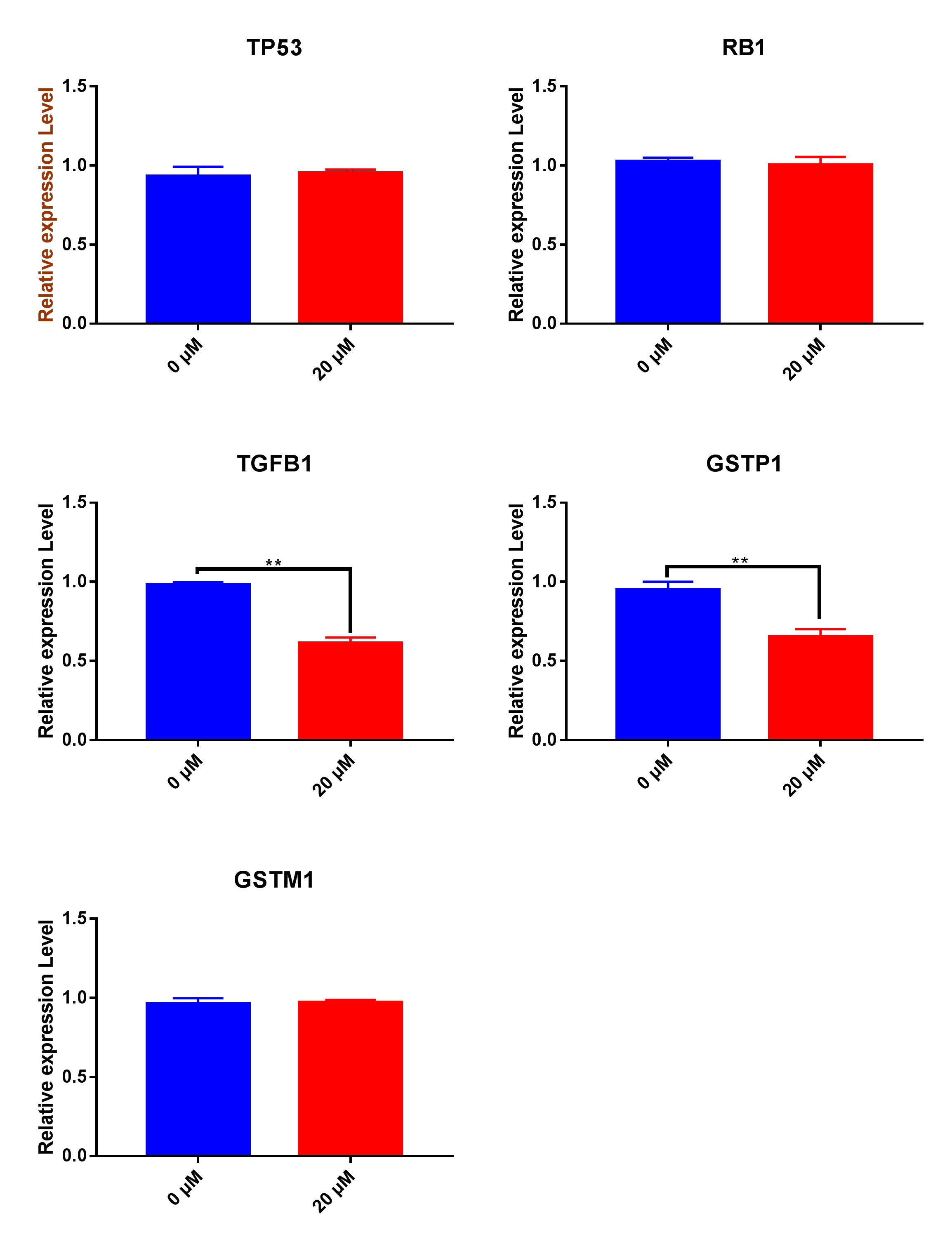

Supplement: Supplemental Information 2 [file peerj-09-12339-s002.zip › Raw data materials/Raw data for figure 4D/Hep3B.tif]

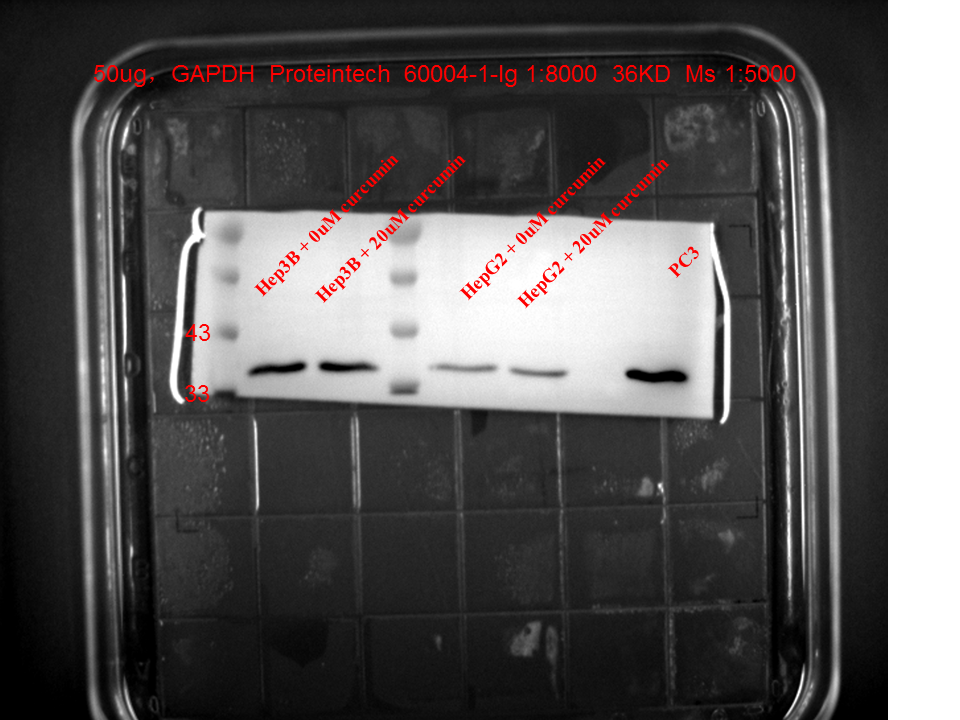

Supplement: Supplemental Information 2 [file peerj-09-12339-s002.zip › Raw data materials/Raw data for figure 4E、F、G、H/GAPDH.PNG]

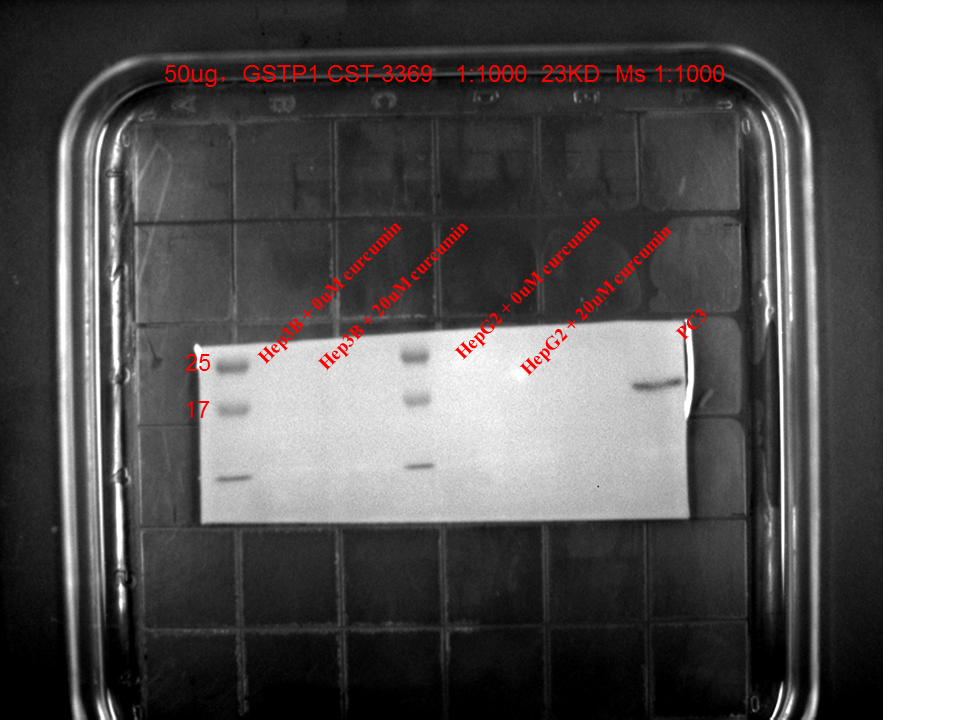

Supplement: Supplemental Information 2 [file peerj-09-12339-s002.zip › Raw data materials/Raw data for figure 4E、F、G、H/GSTP1.PNG]

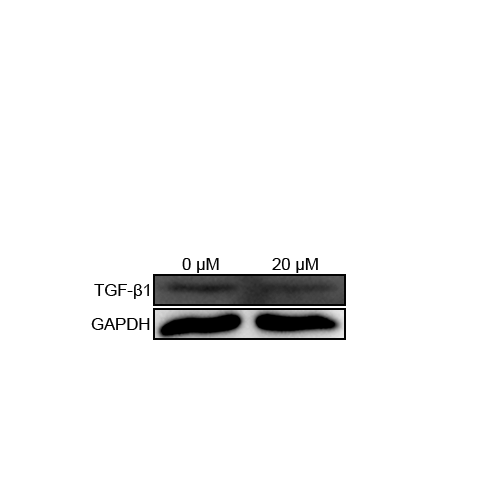

Supplement: Supplemental Information 2 [file peerj-09-12339-s002.zip › Raw data materials/Raw data for figure 4E、F、G、H/Hep3B/TGF-β1-Hep3B.tif]

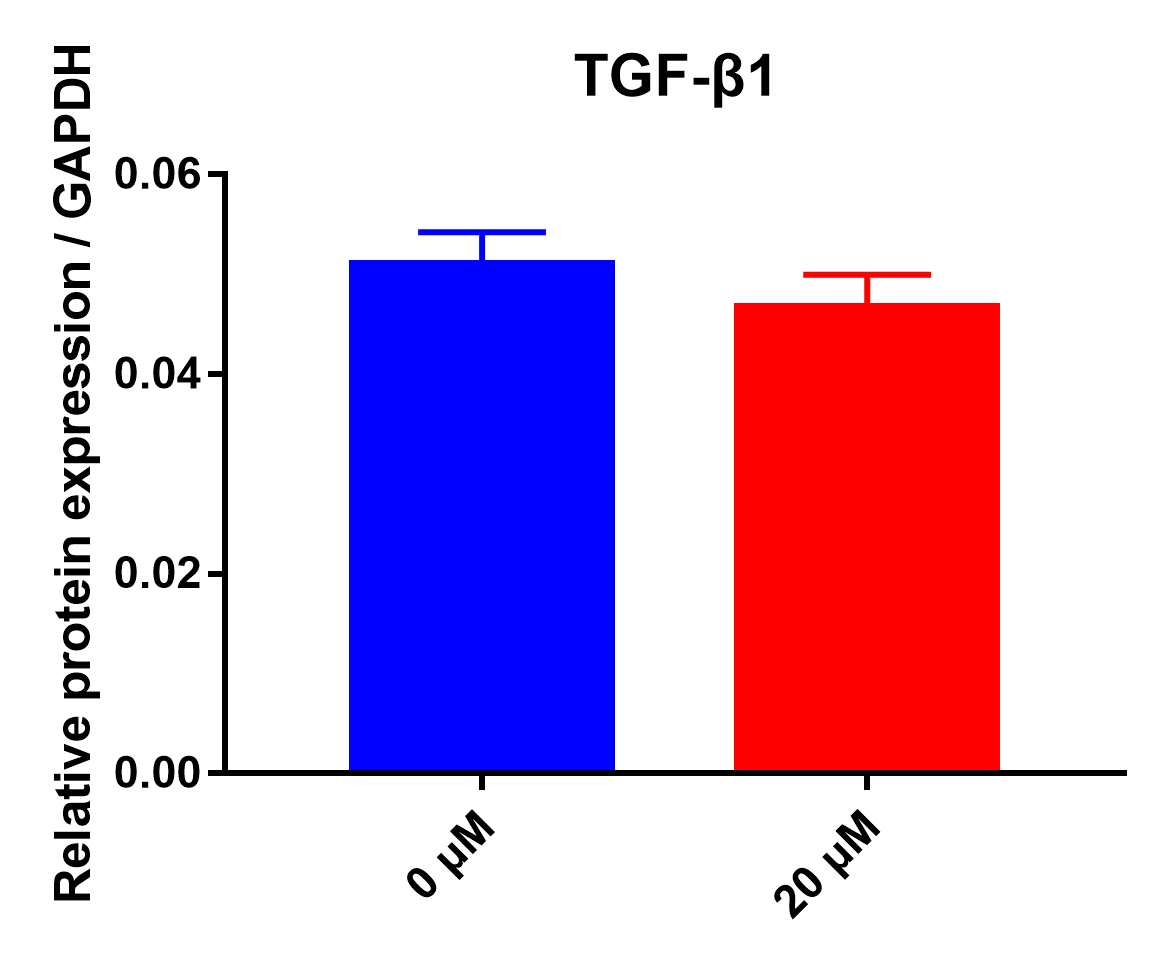

Supplement: Supplemental Information 2 [file peerj-09-12339-s002.zip › Raw data materials/Raw data for figure 4E、F、G、H/Hep3B/TGF-β1_GAPDH-Hep3B.tif]

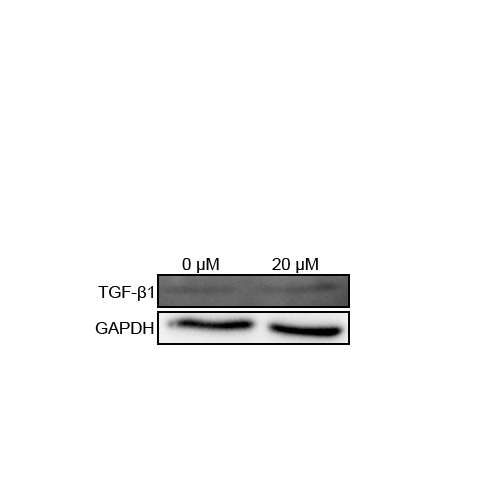

Supplement: Supplemental Information 2 [file peerj-09-12339-s002.zip › Raw data materials/Raw data for figure 4E、F、G、H/HepG2/TGF-β1-HepG2.tif]

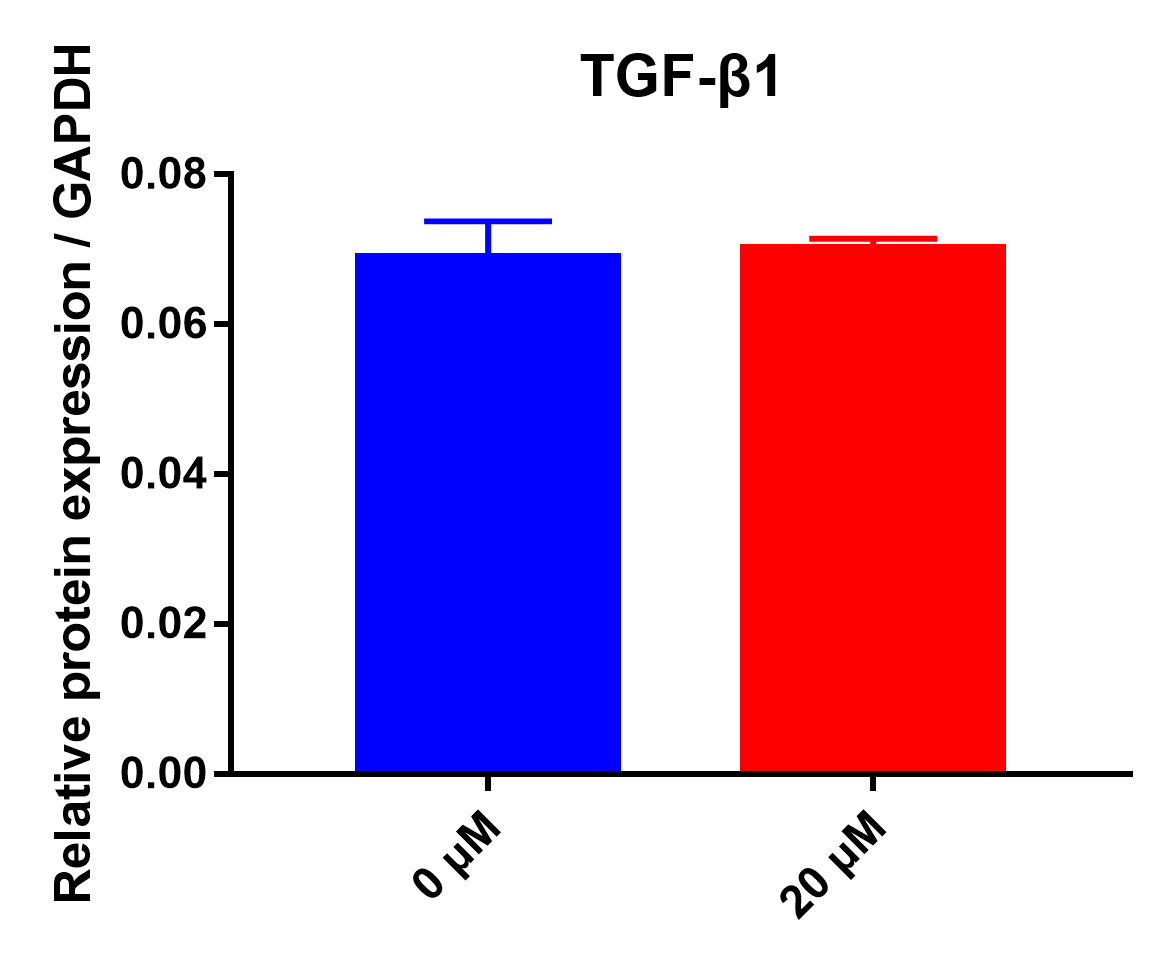

Supplement: Supplemental Information 2 [file peerj-09-12339-s002.zip › Raw data materials/Raw data for figure 4E、F、G、H/HepG2/TGF-β1_GAPDH-HepG2.tif]

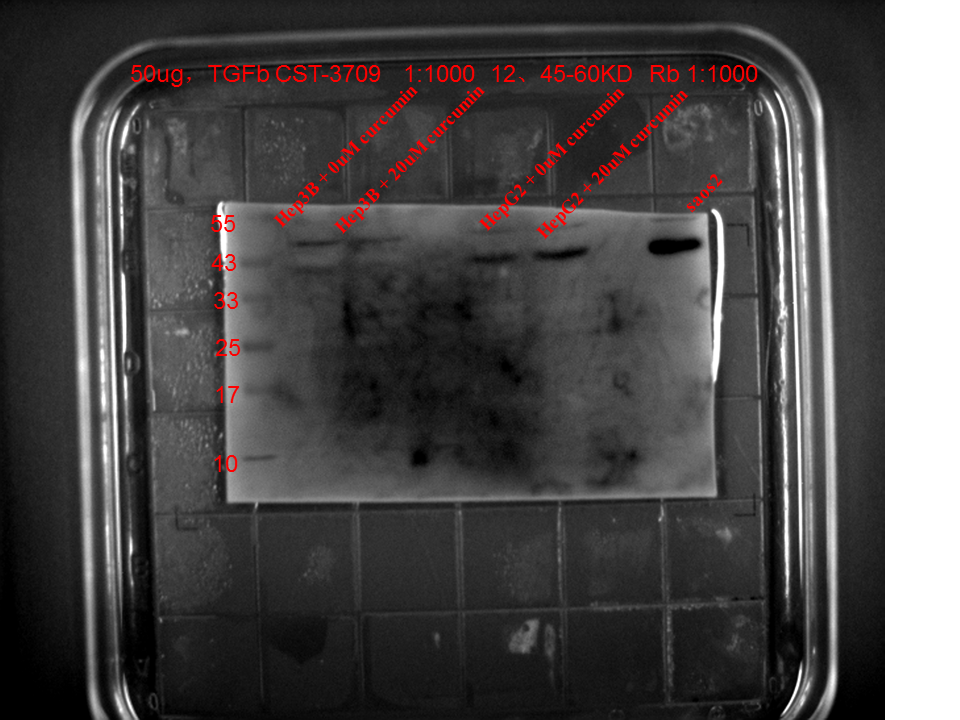

Supplement: Supplemental Information 2 [file peerj-09-12339-s002.zip › Raw data materials/Raw data for figure 4E、F、G、H/TGF-β1.PNG]

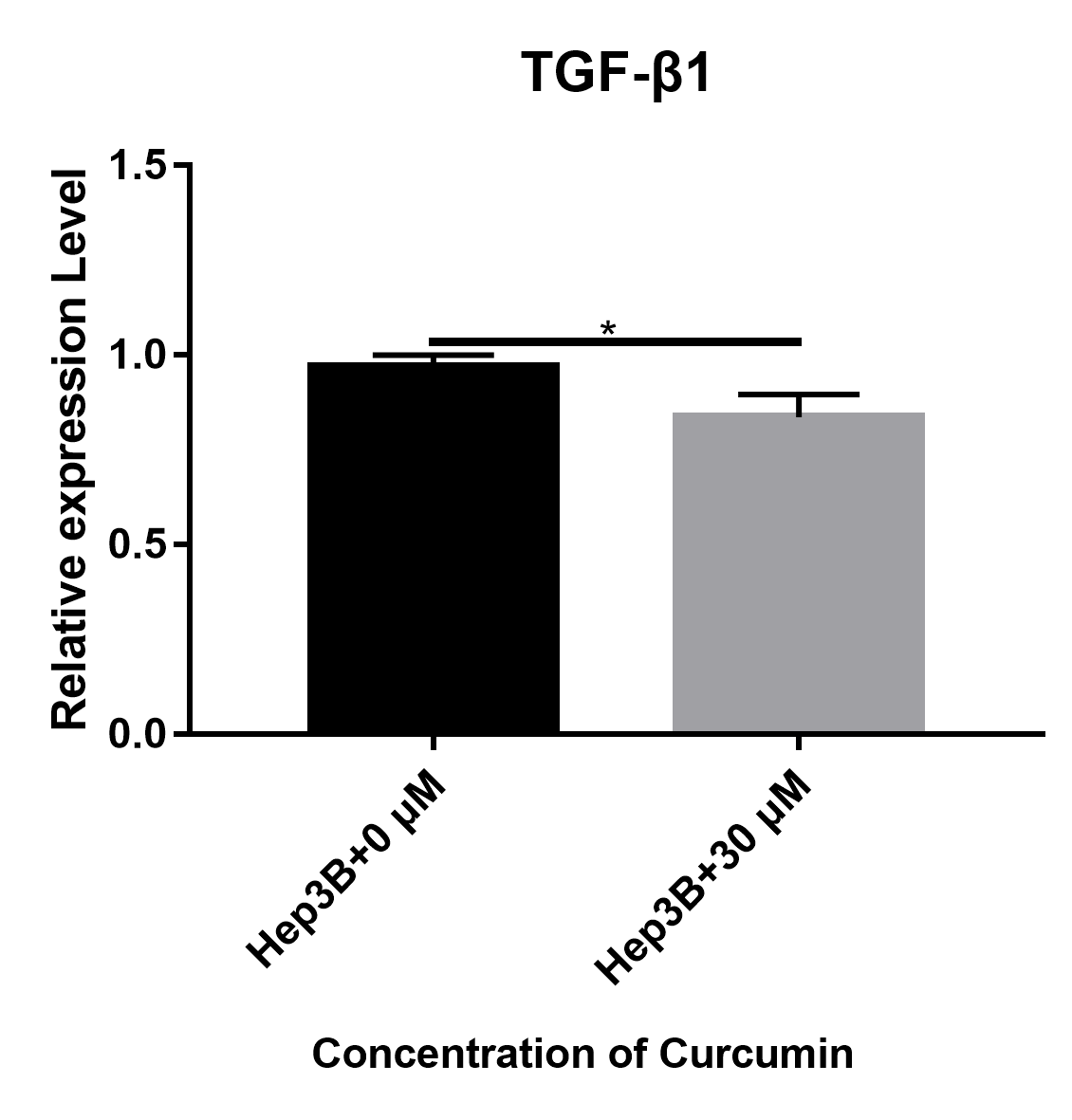

Supplement: Supplemental Information 2 [file peerj-09-12339-s002.zip › Raw data materials/Raw data of PCR analysis in 30uM curcumin/qPCR/TGFB1(Hep3B).tif]

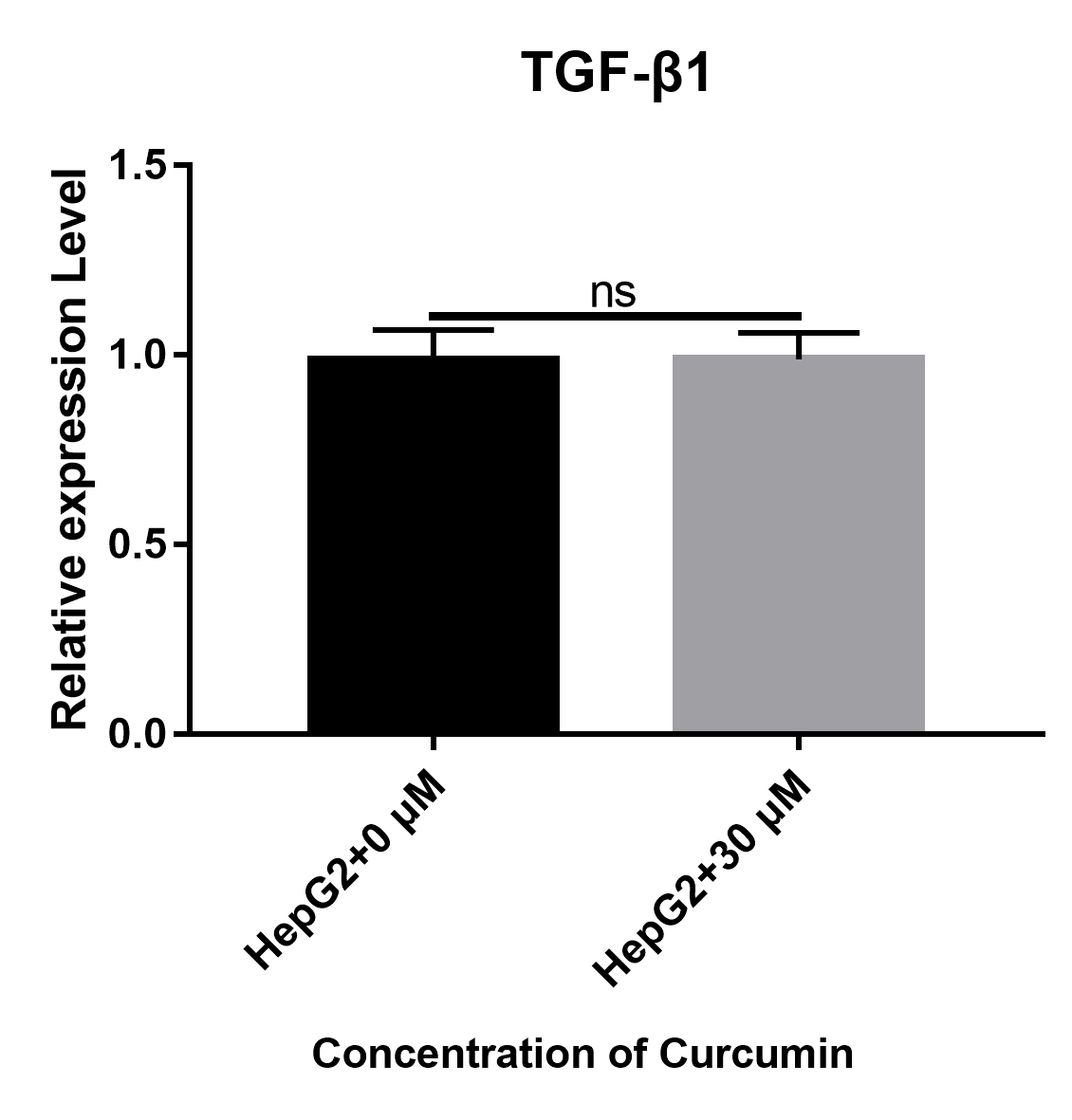

Supplement: Supplemental Information 2 [file peerj-09-12339-s002.zip › Raw data materials/Raw data of PCR analysis in 30uM curcumin/qPCR/TGFB1(HepG2).tif]
